# Dabigatran, Rivaroxaban, or Apixaban versus Warfarin in Patients with Nonvalvular Atrial Fibrillation: A Systematic Review and Meta-Analysis of Subgroups

**DOI:** 10.1155/2013/640723

**Published:** 2013-12-22

**Authors:** Antonio Gómez-Outes, Ana Isabel Terleira-Fernández, Gonzalo Calvo-Rojas, M. Luisa Suárez-Gea, Emilio Vargas-Castrillón

**Affiliations:** ^1^Division of Pharmacology and Clinical Evaluation, Medicines for Human Use, Spanish Agency for Medicines and Medical Devices (AEMPS), c/Campezo 1, Edificio 8, 28022 Madrid, Spain; ^2^Department of Clinical Pharmacology, Hospital Clínico San Carlos, c/Prof. Martín Lagos s/n, 28040 Madrid, Spain; ^3^Department of Pharmacology, Universidad Complutense, Plaza Ramón y Cajal s/n, Ciudad Universitaria, 28040 Madrid, Spain; ^4^Department of Clinical Pharmacology, Hospital Clínic, University of Barcelona, c/Villarroel 170, 08036 Barcelona, Spain

## Abstract

*Background*. New oral anticoagulants (NOAC; rivaroxaban, dabigatran, apixaban) have become available as an alternative to warfarin anticoagulation in non-valvular atrial fibrillation (NVAF). *Methods*. MEDLINE and CENTRAL, regulatory agencies websites, clinical trials registers and conference proceedings were searched to identify randomised controlled trials of NOAC versus warfarin in NVAF. Two investigators reviewed all studies and extracted data on patient and study characteristics along with cardiovascular outcomes. Relative risks (RR) and 95% confidence intervals (CI) were estimated using a random effect meta-analysis. *Results*. Three clinical trials in 50,578 patients were included. The risk of non-hemorrhagic stroke and systemic embolic events (SEE) was similar with the NOAC and warfarin (RR = 0.93; 95% CI = 0.83–1.04), while the risk of intracranial bleeding (ICB) with the NOAC was lower than with warfarin (RR = 0.46; 95% CI = 0.33–0.65). We found differences in the effect size on all strokes and SEE depending on geographic region as well as on non-hemorrhagic stroke, SEE, bleeding and mortality depending on time in therapeutic range. *Conclusion*. The NOAC seem no more effective than warfarin for prevention of nonhemorrhagic stroke and SEE in the overall NVAF population, but are generally associated with a lower risk of ICB than warfarin.

## 1. Introduction

Atrial fibrillation (AF) represents the most common sustained cardiac arrhythmia, affecting more than 6 million people in Europe [1, 2]. AF, particularly when it is persistent/permanent, predisposes patients to the development of atrial thrombi, which may embolize to the systemic circulation, being associated with a 4- to 5-fold increase in the risk of ischemic stroke [3].

Vitamin K antagonists (VKA; coumarins, like warfarin and acenocoumarol) have been the only oral anticoagulants available over the last 60 years [4]. These agents are effective to prevent stroke in patients with AF [5], but their management remains problematic due to their narrow therapeutic index and variability in drug exposure, necessitating routine coagulation monitoring (international normalised ratio (INR)), clinical surveillance, and continuous patient education [6]. As a result, approximately only half of eligible patients with AF receive oral anticoagulation with VKA [7].

Dabigatran etexilate (Pradaxa, Boehringer Ingelheim) [8], rivaroxaban (Xarelto, Bayer HealthCare) [9], and apixaban (Eliquis, Bristol Myers Squibb) [10] are new oral anticoagulants (NOAC) available in Europe and other countries. Unlike VKA, these new compounds exhibit a predictable dose response and do not require routine coagulation monitoring, but their anticoagulant effect declines quickly in case of poor compliance, and not coagulation monitoring tests or specific antidotes are currently available [4]. Among other indications, these new compounds have been tested for prophylaxis of stroke and systemic embolic events (SEE) in patients with nonvalvular atrial fibrillation (NVAF) using a combined primary outcome of all strokes (nonhemorrhagic and haemorrhagic) and SEE. The pivotal studies conducted in that indication are usually large clinical trials recruiting heterogeneous populations in different geographic regions [11]. The potential influence of differences in clinical, demographic, and geographic factors across studies on the relative efficacy and safety of the NOAC, as well as the clinical relevance of these differences, has not been thoroughly analysed. For that purpose, we systematically reviewed the data from randomised controlled trials with the NOAC for prevention of stroke and SEE in patients with NVAF.

## 2. Methods

### 2.1. Study Selection

We searched MEDLINE and CENTRAL (up to 31 December 2012), clinical trial registries, relevant conference proceedings, and regulatory agencies websites (see supplementary file for search strategy in Supplementary Material available at http://dx.doi.org/10.1155/2013/640723). No language restrictions were applied.

To maximize the real-world applicability of our results to relevant subgroups, we considered randomised phase III controlled trials with available subgroup analyses comparing any of the approved NOAC (i.e., rivaroxaban, dabigatran, and apixaban) with warfarin for prevention of stroke and SEE in patients with NVAF. At least one of the daily doses tested in the experimental arms had to correspond to the approved regime for the NOAC: dabigatran 150 or 110 mg twice daily (BID), rivaroxaban 20 mg once daily (OD), and apixaban 5 mg BID. At least one of the drug control groups had to correspond to warfarin, dose adjusted to achieve a target INR of 2 to 3.

### 2.2. Data Extraction

Two investigators (Antonio Gómez-Outes and Ana Isabel Terleira-Fernández) independently and separately assessed trials for eligibility and extracted data. If a trial was covered in more than one report we used a hierarchy of data sources: public reports from regulatory authorities (US Food and Drug Administration, European Medicines Agency), peer-reviewed articles, reports from the web based repository for results of clinical studies, and other sources.

The following study characteristics were collected: number of patients; dosage of the experimental and control groups; trial phase and design; inclusion and exclusion criteria; main efficacy and safety outcomes; main populations and period of analyses; definition of noninferiority; adjudication committees of clinical events; median length followup; and time in therapeutic range (TTR). We assessed study quality using the Jadad scale [12]. Additionally, we used the Cochrane Collaboration's tool for assessing risk of bias in randomised studies [13].

The prespecified primary efficacy outcome was nonhemorrhagic (ischemic/undefined) stroke and SEE [14]. The main safety outcome was intracranial bleeding (ICB; composite of hemorrhagic stroke, subdural, subarachnoid, or epidural hemorrhage) [14]. A net clinical outcome was included as the composite of all strokes and SEE, which was consistent with the primary efficacy outcome in individual trials and in previous publications assessing the net clinical benefit of anticoagulant therapy [14]. A subanalysis of all strokes and SEE occurring after study drug discontinuation (i.e., temporary interruptions, permanent discontinuation, and after end of study) was also conducted. Other secondary outcomes included the components of the main efficacy and safety endpoints as well as major bleeding, major gastrointestinal bleeding, and mortality.

We collected outcome data on the overall trial populations as well as on the following 12 relevant subgroups: geographic region (Europe or other regions), prior stroke or transient ischemic attack (yes or no), quality of warfarin therapy (TTR ≥ 65% or <65%), degree of thromboembolic risk by applying the CHADS^2^ score (≥2 or <2 score points), age (≥75 yrs or <75 yrs), gender (male or female), diabetes (yes or no), heart failure (yes or no), renal function (creatinine clearance ≥50 mL/min or <50 mL/min), type of AF (permanent/persistent or paroxysmal), prior use of VKA (yes or no), and concomitant use of acetylsalicylic acid (yes or no). Finally, we contacted sponsors or the main investigators for missing outcome data of interest.

### 2.3. Statistical Analysis

We carried out direct comparisons between the NOAC and warfarin on an intention-to-treat basis, according to PRISMA (Preferred Reporting Items for Systematic Reviews and Meta-Analyses) recommendations [15].

For the meta-analysis we calculated relative and absolute risks and their respective 95% confidence intervals (CI) for each study and for the pooled studies. Heterogeneity was assessed using the Cochran *Q* test [16] and the Higgins *I* ^2^ test [17]. A Cochran's *Q* *P* < 0.10 and *I* ^2^ > 50% were considered to show significant heterogeneity between studies or subgroups [17]. We used the random effects model described by DerSimonian and Laird for the main analysis [18]. We carried out subgroup analyses according to relevant clinical characteristics (see “Outcome measures”). Pooled absolute risk estimates (obtained using risk difference meta-analysis) were annualized taking into account median followup during the studies. We also calculated the number needed to treat to benefit (NNTB) or to harm (NNTH) (as the inverse of the absolute risk difference) and corresponding confidence intervals for main outcomes [19]. We performed sensitivity analyses using the fixed effects method described by Mantel and Haenszel [20] and including only studies at low risk of bias. Baseline characteristics were compared across studies using Chi-square test for categorical variables and one-way analysis of variance (ANOVA) for continuous variables. Statistical calculations were done using the RevMan statistical software, version 5.1 (Nordic Cochrane Center) [21], and Microsoft Office Excel 2003 (Redmond, Washington; Microsoft Corporation, 2003).

## 3. Results

### 3.1. Descriptive Analysis

**Figure 1:**
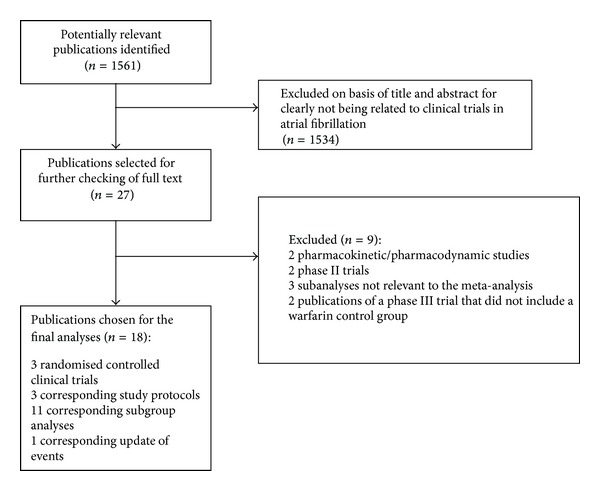
Study identification, selection, and exclusions.

The literature search identified 1561 articles, 27 of which related to clinical trials or protocols with rivaroxaban, dabigatran, or apixaban in AF (Figure 1) and were selected for checking as full text. Three articles of phase III clinical trials with dabigatran (RE-LY study) [22], rivaroxaban (ROCKET-AF study) [23], and apixaban (ARISTOTLE study) [24] and their corresponding protocols [25–27] were eligible for inclusion. We also included 11 subanalyses of these trials that were considered relevant for the meta-analysis [28–38] and 1 article corresponding to update of events [39] reported in the RE-LY study [22]. The remaining 9 articles did not meet inclusion criteria and were excluded [40–48]. We also identified five public reports from the US Food and Drug Administration website [49–53] that included supplementary data of the RE-LY, ROCKET-AF, and ARISTOTLE studies, as well as one additional analysis of posttreatment events in the ROCKET-AF study [54] and one additional subanalysis of the ARISTOTLE study [55].

Table 1 shows the characteristics of the trials and treatments. The 3 studies comprised 50,578 patients and compared dabigatran (*n* = 12,091) [22], rivaroxaban (*n* = 7131) [23], or apixaban (*n* = 9120) [24] with warfarin (*n* = 22,236) [22–24]. In RE-LY, dabigatran was administered at fixed twice-daily doses of 150 mg or 110 mg [22]. On the contrary, in ROCKET-AF, the rivaroxaban 20 mg OD dose had to be down-titrated to 15 mg in patients with moderate renal impairment [23], while in ARISTOTLE, the apixaban 5 mg BID dose had to be down-titrated to 2.5 mg BID for patients with two or more of the following criteria: age ≥ 80 years, body weight ≤ 60 kg, and/or serum creatinine level ≥ 1.5 mg per decilitre [24]. RE-LY scored 3 points on the Jadad scale and was considered to be at moderate risk of bias because it was an open study [22]. ROCKET-AF and ARISTOTLE were double-blind trials [23, 24], scoring 5 points on the Jadad scale, and were judged to be at low risk of bias. The risk-of-bias assessment following Cochrane recommendations [13] showed similar results, with RE-LY [22] considered to be at unclear risk of bias and ROCKET-AF [23] and ARISTOTLE [24] judged to be at low risk of bias (see supplementary Table A1).

Table 2 shows the characteristics of patients and events rates in the warfarin control group in each study. Median age ranged between 70 and 73 years across trials, with a predominance of male sex. However, other characteristics, like percentage of patients with CHADS ≥ 2, prior stroke/transient ischemic attack, congestive heart failure, age > 75 years, type of AF, prior use of VKA or acetylsalicylic acid, and percent population enrolled in Europe, were widely heterogeneous. Demographic data were also compared for patients with prior stroke or transient ischemic attack (see supplementary Table A2) and a similar heterogeneity was found.

### 3.2. Primary Efficacy Outcome

**Figure 2:**
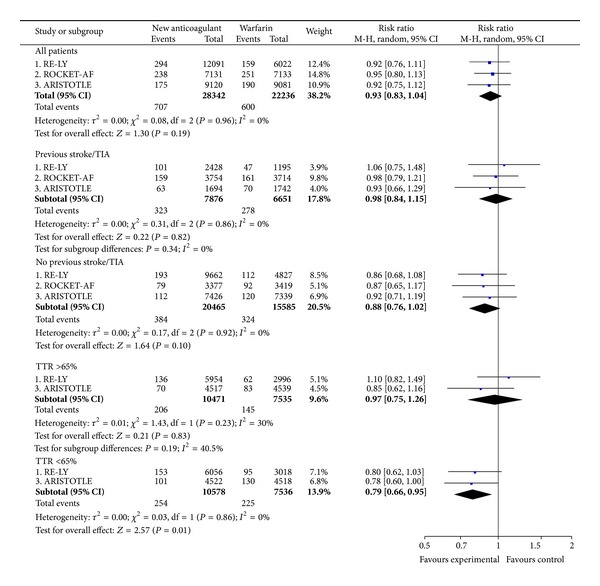
Nonhemorrhagic stroke and systemic embolic events.

The NOAC were not more effective than warfarin in preventing nonhemorrhagic stroke and SEE in the overall study populations (RR = 0.93; CI 0.83 to 1.04) (Figure 2). However, subgroup analyses suggest a trend towards superiority of the NOAC in centres with TTR <65% (Figure 2). Approximately 92% of the events were nonhemorrhagic strokes and only 8% were SEE. The separate results for nonhemorrhagic stroke (RR = 0.95; CI 0.85 to 1.07) and SEE (RR = 0.73; CI 0.50 to 1.07) were consistent with those of the composite endpoint. The full subgroup analyses of the primary efficacy outcome are included in supplementary Figure A1.

### 3.3. Primary Safety Outcome

**Figure 3:**
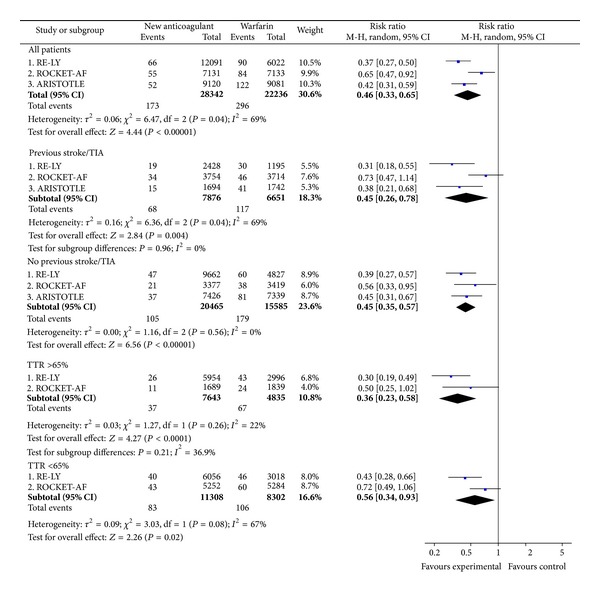
Intracranial bleeding.

The NOAC reduced the relative risk of ICB in comparison with warfarin (RR = 0.46; CI 0.33 to 0.65) (Figure 3). However, there was a significant heterogeneity between the three studies due to a lower reduction of ICB by rivaroxaban in the ROCKET-AF study than by dabigatran or apixaban in the other studies (*P* = 0.04; *I* ^2^ = 69%) (Figure 3). The poorer effect of rivaroxaban versus warfarin on ICB was mainly observed in patients with prior stroke (Figure 3) and should be interpreted in line with the much lower rate of ICB with warfarin in this subpopulation of the ROCKET-AF study compared with the corresponding subpopulations of the RE-LY and ARISTOTLE studies (ROCKET-AF 1.24% versus RE-LY 2.51% versus ARISTOTLE 2.35%; *P* = 0.0099) (see supplementary Table A2). Approximately 60% of the ICB were hemorrhagic strokes and 40% corresponded to other types of ICB (e.g., subdural, subarachnoid, and epidural). The separate results for hemorrhagic stroke (RR = 0.45; CI 0.30 to 0.66) and other types of ICB (RR = 0.47; CI 0.27 to 0.82) were consistent with those of the composite endpoint.

### 3.4. Secondary Outcomes

#### 3.4.1. All Strokes and Systemic Embolic Events (Intention-to-Treat)

**Figure 4:**
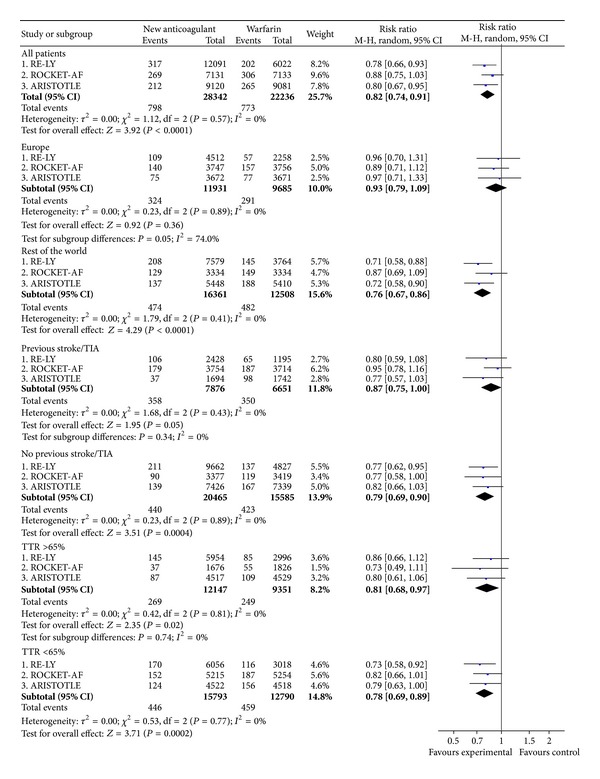
All strokes and systemic embolic events (intention-to-treat).

In the overall study populations, the NOAC afforded a lower relative risk of events than warfarin (RR = 0.82; CI 0.74 to 0.91) (Figure 4). However, there were significant geographic differences. Within Europe, the NOAC did not significantly reduce the rates of events compared with warfarin, while outside of Europe the NOAC appeared superior in this regard (Figure 4). The full subgroup analyses of all strokes or SEE are included in supplementary Figure A3.

#### 3.4.2. All Strokes and Systemic Embolic Events after Treatment

**Figure 5:**
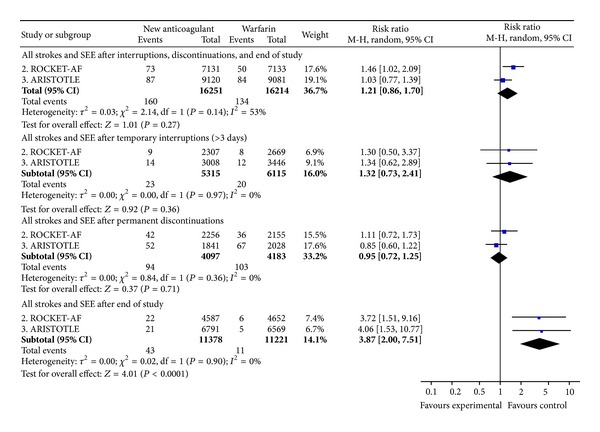
All strokes and systemic embolic events after study drug discontinuation.

There was no statistically significant difference between the NOAC and warfarin in the risk of stroke and SEE after temporary interruptions or permanent discontinuations (Figure 5). However, after end of study, patients who were transitioned from the NOAC to warfarin experienced significantly more events within the first 30 days of transition than patients on the warfarin group (RR = 3.87; CI 2.00 to 7.51) (Figure 5). The vast majority of events corresponded to ischemic/undefined stroke.

#### 3.4.3. Major Bleeding and Deaths

**Figure 6:**
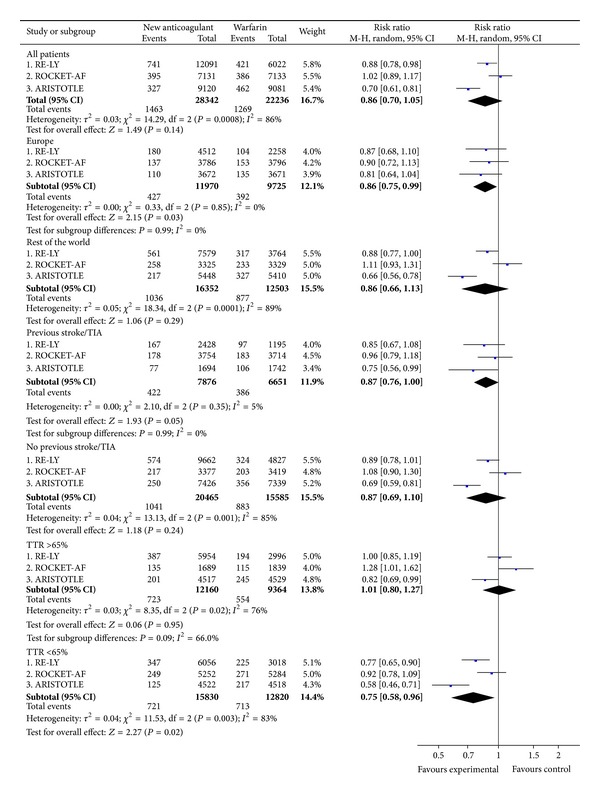
Major bleeding.

There was a trend towards lower rates of major bleeding with the NOAC in comparison with warfarin in RE-LY and ARISTOTLE and neutral in ROCKET-AF (Figure 6). The trend towards reduction in major bleeding rates was homogeneous in patients with previous stroke or transient ischemic attack (RR = 0.87; CI 0.76 to 1.00) and in patients recruited in Europe (RR = 0.86; CI 0.75 to 0.99) (Figure 6). Between-subgroups significant heterogeneity was shown depending on TTR (*P* = 0.09; *I* ^2^ = 66%), with better results of the NOAC on major bleeding in centres with TTR < 65% than in centres with TTR ≥ 65% (Figure 6). The full subgroup analyses of major bleeding are included in supplementary Figure A4.

**Figure 7:**
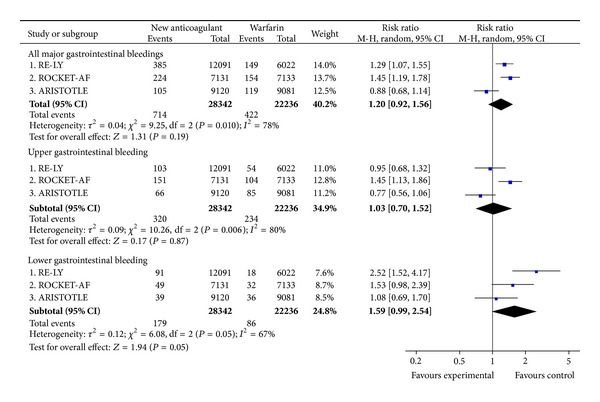
Major gastrointestinal bleeding.

There was a trend towards higher rates of major gastrointestinal bleeding with the NOAC in comparison with warfarin in RE-LY and ROCKET-AF and no increase in ARISTOTLE (Figure 7). Analysing the site of bleeding, rivaroxaban showed a trend towards increased risk of major upper gastrointestinal bleeding and major lower gastrointestinal bleeding, while dabigatran increased the risk of major lower gastrointestinal bleeding (Figure 7).

**Figure 8:**
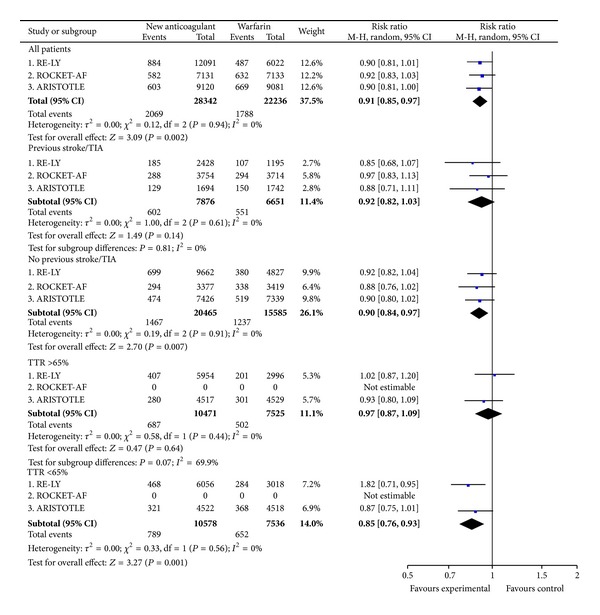
Mortality.

Overall, the NOAC reduced the risk of death in comparison with warfarin (Figure 8). However, the effect on death was heterogeneous depending on the TTR (*P* = 0.07; *I* ^*2*^ = 69.9%), being nonsignificant in centres with TTR ≥65% (RR = 0.97; CI 0.87 to 1.09) and significant in centres with TTR <65% (RR = 0.85; CI 0.76 to 0.93) (Figure 8). The full subgroup analyses of death are included in supplementary Figure A5.

### 3.5. Absolute Difference in Events per 1000 Patients Treated

Compared with warfarin, the NOAC did not avoid a significant number of nonhemorrhagic strokes or SEE events per 1000 patients treated in the overall study populations (−1; CI −2.4 to 0.5) (Table 3). However, the reduction was significant in centres with TTR <65% (−3.5; CI −6.3 to −0.8) (Table 3).

The NOAC avoided a significant number of ICB in the overall study populations (−3.7; CI −5.3 to −3.1) (NNTB per year, 271; CI 190 to 469), which was particularly relevant in patients with prior stroke or SEE (−5.8; CI −10.9 to −2.3) (NNTB per year, 173; CI 92 to 437) (Table 3).

The numbers of all strokes and SEE avoided per 1000 patients per year were highly dependent on the geographic region and quality of warfarin therapy, with no significant differences in European patients or in centres with TTR ≥65%. The absolute risk reduction in major bleedings was mainly observed in centres with TTR <65% (−7.1; CI −12.7 to −1.6) (Table 4).

The absolute risk difference in all-cause death per 1000 patients treated per year (−3.90; CI −6.34 to −1.40) was highly dependent on quality of warfarin therapy. No significant differences between the NOAC and warfarin were seen in centres with TTR ≥65% (−0.9; −4.8 to 2.9), while a significant absolute risk reduction was achieved in centres with TTR <65% (−6.9; CI −11.2 to −2.6).

### 3.6. Sensitivity Analyses

Sensitivity analyses using the fixed effects model and including only studies at low risk of bias were consistent with the main analysis (see supplementary Table A3).

### 3.7. Role of Funding

All studies were sponsored by pharmaceutical companies. In all cases, the sponsor was involved in the study design and oversight with the collaboration of a research institute and a scientific committee. In RE-LY [22], the Population Health Research Institute (Hamilton, ON, Canada) independently managed the database and performed the primary data analyses. In ROCKET-AF [23], the Duke Clinical Research Institute (Durham, NC, USA) coordinated the trial, managed the database, and performed the primary analyses independently of the sponsors. In ARISTOTLE [24], the primary analyses were performed both at Bristol-Myers Squibb and at the Duke Clinical Research Institute. At least one of the authors of the publications was employee of the sponsor.

## 4. Discussion

This systematic review, comprising more than fifty thousand patients enrolled in 3 randomised clinical trials, is to our knowledge the first systematic attempt to assess separately the efficacy of the NOAC in preventing thromboembolic events (nonhemorrhagic stroke and SEE) and major prohemorrhagic effects (ICB) [14] in NVAF. The data indicate that the NOAC have a generally similar efficacy than warfarin in the prevention of nonhemorrhagic stroke and SEE. This efficacy endpoint, which does not include hemorrhagic stroke, differs from the main outcome chosen for pivotal trials with NOAC in NVAF, which has been a net clinical endpoint including all strokes (ischemic, hemorrhagic, or undefined/unknown type) and SEE [22–24]. Our results are not inconsistent with the primary efficacy analyses of the respective studies but clearly suggest that, in the overall population, the weight of the effect tends to rely on the reduction of ICB, rather than on the antithromboembolic effect.

Two relevant meta-analyses of the NOAC in NVAF have been recently published [56, 57]. These meta-analyses showed an overall clinical benefit of the NOAC versus warfarin in NVAF, which is consistent with the results of our meta-analysis regarding the net clinical endpoint of all strokes and SEE. With respect to the assessment of efficacy, there are some methodological differences between our meta-analysis and those conducted by Dentali et al. [56] and Miller et al. [57]. We analysed the composite of ischemic/undefined strokes and SEE, while the other meta-analyses only included ischemic strokes [56] or ischemic/undefined strokes [57]. Notwithstanding, the meta-analysis by Dentali et al. showed a similar efficacy of the NOAC and warfarin in preventing ischemic stroke (RR = 0.92; CI 0.81 to 1.04) [56], which is consistent with the similar efficacy in preventing nonhaemorrhagic stroke and SEE found in our meta-analysis. On the other hand, the meta-analysis by Miller et al. [57] included the dabigatran 150 mg BID dose but excluded the dabigatran 110 mg BID dose, as the meta-analysis was conducted from a US perspective and the dabigatran 110 BID dose is not currently approved in the US for use in NVAF. This issue could result in an overestimation of the efficacy of the NOAC versus warfarin in preventing ischemic/undefined strokes in their meta-analysis (RR = 0.87; CI 0.77 to 0.99) [57]. On the contrary, we included both dabigatran doses (150 mg BID and 110 mg BID), because both are already approved in Europe and many other regions for use in NVAF. The long-term extension of the RE-LY study has shown no differences in efficacy between the high and low dabigatran dose in the long term [58], which further supports our decision of including both dabigatran doses in the meta-analysis.

To the best of our knowledge, our systematic review is the first one that analyses specific subgroups and gives absolute risks estimates, thus providing a clear picture about the absolute benefit in efficacy or safety that may be expected with the NOAC in the heterogeneous population of patients with AF.

Although this review shows that the overall net clinical benefit of the NOAC versus warfarin is favourable, the magnitude of such benefit may be however influenced by a number of factors, as suggested by subgroup analyses. In RE-LY and ARISTOTLE, superiority in the composite of all strokes and SEE was mainly gained at expenses of events that occurred in non-European countries (e.g., South America, Asia, and Africa), while all the NOAC were consistently not superior to warfarin in Europe. In the ROCKET study, with a higher proportion of European patients, these differences were not apparent. It is hard to believe that geography itself influences treatment effect, but it may influence the way patients are managed in clinical practice [59, 60]. Potential interaction factors accounting for geographic differences may comprise the quality of oral anticoagulation and control of associated risk factors for thrombosis (e.g., hypertension, diabetes, and heart failure). The benefit of oral anticoagulation is largely dependent on the quality of INR control achieved by centers and countries as measured by TTR [61, 62]. The use of center-based TTRs as a proxy for individual-level INR control is a matter of controversy, but it may be considered a reasonable approach in clinical trials comparing the NOAC and warfarin in AF [28]. Individual-level comparisons between the NOAC and warfarin would increase the relevance of the results to decision-making, but these comparisons are very difficult to conduct given that there were no comparable INRs in the treatment arms with the NOAC. While the understanding of the determinants of individual TTR remains incomplete, it is clear that the providers of care, and the systems within which they work, have a profound effect on the quality of anticoagulation [63]. Beyond statistical significance, subgroup analyses suggest that the net benefit of the NOAC seems better than that of warfarin in situations in which quality of oral anticoagulation is poor, given that thromboembolic complications, major bleeding, and mortality may be decreased, as well as in patients with prior stroke or transient ischemic attack, particularly if they have concomitant predictive factors for developing ICB (e.g., leukoaraiosis) [64], given that the absolute risk reduction in ICB may be significant.

Switching between anticoagulants may be sometimes clinically indicated and have deleterious consequences if not properly planned. In ROCKET-AF and ARISTOTLE, an excess in (mainly ischemic) strokes occurred in the rivaroxaban and apixaban arms upon discontinuation at the end of the trial. This resumption of events was probably related to inadequate control of anticoagulation, but induction of a hypercoagulable state by long-term treatment with the NOAC has not been ruled out [51].

The present systematic review has limitations. The main efficacy outcome in our study (nonhemorrhagic stroke and SEE) was a part of the main net clinical outcome (all strokes and SEE) in individual studies. In addition, the main safety outcome in our study (ICB) was a secondary safety endpoint in individual studies. On the other hand, we conducted subgroup analyses, which have well-known limitations [65]. Testing multiple subgroups, even though prespecified, creates the possibility of false-positive findings. However, when subgroups are described in the protocol of the original trials along with a stated hypothesis, these secondary analyses may be used to illustrate applicability across patient subgroups [65, 66]. RE-LY, ROCKET-AF, and ARISTOTLE trials included a heterogeneous population (Table 2), and in such a situation, subgroup analyses are reasonable. In addition, most subgroups included a significant number of patients (e.g., 14,527 had prior stroke/TIA and 21,695 were recruited in European centers; Table 2) thus having sufficient statistical power to detect clinically meaningful differences between treatments.

At the time of translating the results from these clinical trials into practise, some additional considerations are necessary. On the one hand, thrombotic and haemorrhagic events in the real-world anticoagulated AF population are higher than those reported in clinical trials [67, 68], probably due to the strict selection of population and close followup applied in clinical trials. In particular, patients aged 75 years or older were underrepresented in clinical trials (range: 31% to 43% of patients) compared with real-world AF cohorts (range: 47% to 64% of patients) [69]. This issue may have important implications in bleeding risk, as renal function declines with age and all NOAC undergo renal elimination to a greater or lesser extent. Postmarketing reports of serious bleedings have frequently involved patients generally not qualified for the NOAC (i.e., severe renal insufficiency) [70]. This finding, accompanied by the current unavailability of specific antidotes, emphasizes the need for their appropriate use according to product labelling in order to minimise bleeding risk [8–10].

Finally, there is a need for strategies that could optimize anticoagulation quality and improve clinical outcomes in AF [71]. Beyond the use of NOAC in selected patients, these strategies may include systems facilitating algorithm-based warfarin dosing in the anticoagulation clinics [72], as well as the use of home-monitoring and self-management of anticoagulation with VKA in suitable candidates [73].

## 5. Conclusion

The NOAC seem no more effective than warfarin in preventing nonhemorrhagic stroke and SEE in NVAF. However, they are generally associated with a lower risk of ICB than warfarin. The net benefit of the NOAC seems better than that of warfarin in situations in which quality of oral anticoagulation is poor, given that thromboembolic complications, major bleeding, and mortality may be decreased, as well as patients with prior stroke or transient ischemic attack, as the absolute risk reduction in ICB may be particularly significant. However, the absolute benefit of the NOAC tends to be of a lesser magnitude in Europe than in other regions, which might be due to regional differences in quality of oral anticoagulation and overall management of associated risk factors for thrombosis. These findings would deserve further investigation.

## Supplementary Material

Supplementary Material includes details of the bibliographic search, as well as risk of bias assessment for the included studies, characteristics of the patients and events in patients with prior stroke or transient ischemic attack, sensitivity analyses depending on statistical model used and risk of bias, and complete subgroup analyses for main and key secondary outcomes.

## Figures and Tables

**Table 1:** Characteristics of the studies and treatments.

Drug, trial	Dabigatran RE-LY [22, 48]	Rivaroxaban ROCKET [23, 49]	Apixaban ARISTOTLE [24, 50]
No. in sample	18113	14264	18201
Treatment characteristics			
Experimental drug	Dabigatran 150 mg or 110 mg twice daily	Rivaroxaban 20 mg or 15 mg once daily	Apixaban 5 mg or 2.5 mg twice daily
Experimental, *n*	12091	7131	9120
High-dose	6076	5624	8702
Low-dose	6015	1597	428
Control drug	Warfarin dose-adjusted to INR 2-3, once daily	Warfarin dose-adjusted to INR 2-3, once daily	Warfarin dose-adjusted to INR 2-3, once daily
Control, *n*	6022	7133	9081
TTR (%)			
Mean	64.4	55.2	62.2
Median	67	58	66
Trial phase	III	III	III
Design of randomised controlled trial	Multicentre, open-label PROBE	Multicentre, double-blind	Multicentre, double-blind
Adjudicating committee and blinded adjudication of outcomes	Yes	Yes	Yes
Interim analysis, *n*	2	1	1
Number of exclusion criteria	14	31	19
Main efficacy outcome	Stroke and SEE	Stroke and SEE	Stroke and SEE
Main analysis	Non-inferiority	Non-inferiority	Non-inferiority
Non-inferiority margin	Relative risk < 1.46	Relative risk < 1.46	Relative risk < 1.38
Main population of analysis	Intent-to-treat	Per protocol	Intent-to-treat
Main period of analysis	Until notification of study termination	On-treatment plus 2 days	Until notification of study termination
Main safety outcome	Major bleeding	Clinically relevant bleeding	Major bleeding
Main population of analysis	Safety population	Safety population	Safety population
Main period of analysis	On-treatment plus 6 days*	On-treatment plus 2 days*	On-treatment plus 2 days*
Jadad Score	3	5	5
Median length follow-up (days)	730	707	657

*After treatment discontinuation.

INR: international normalised ratio; PROBE: prospective, open-label, blinded endpoint; SEE: systemic embolic events; TTR: time in therapeutic range.

**Table 2:** Characteristics of the patients and events (overall study population).

Drug, trial	Dabigatran RE-LY [22, 48]	Rivaroxaban ROCKET [23, 49]	Apixaban ARISTOTLE [24, 50]	*P*-value*
No in sample	18113	14264	18201	
Patients characteristics				
Age (years)	72 (mean)	73 (median)	70 (median)	—
Male gender	11514 (64%)	8604 (60%)	5660 (65%)	<0.0001
CHADS_2_ (mean ± standard deviation)	2.1 ± 1.1	3.46 ± 0.95	2.1 ± 1.1	<0.0001
CHADS_2_ ≥ 2	12337 (68%)	14261 (*≈*100%)	12018 (66%)	<0.0001
CHADS_2_ = 1	5775 (32%)	3 (*≈*0%)	6183 (34%)	<0.0001
Prior stroke/transient ischemic attack	3623 (20%)	7468 (55%)	3436 (19%)	<0.0001
Congestive heart failure	5793 (32%)	8908 (63%)	6451 (35%)	<0.0001
Hypertension	14283 (79%)	12910 (91%)	15916 (87%)	<0.0001
Age ≥ 75 years	7238 (40%)	6229 (43%)	5678 (31%)	<0.0001
Diabetes	4221 (23%)	5695 (40%)	4547 (25%)	<0.0001
Prior myocardial infarction	3005 (17%)	2468 (17%)	2585 (14%)	<0.0001
Patients in centers with TTR ≥ 65%	8950 (49%)	3493 (24%)	9046 (50%)	<0.0001
Patients recruited in Europe	6770 (37%)	7582 (53%)	7343 (40%)	<0.0001
Patients with CrCl < 50 mL/min	3505 (19%)	2986 (21%)	3017 (17%)	<0.0001
Type of atrial fibrillation				
Permanent-persistent	12164 (67%)	11548 (81%)	15412 (85%)	<0.0001
Paroxysmal	5943 (33%)	2514 (18%)	2786 (15%)	<0.0001
Antithrombotic treatment at baseline				
VKA	8989 (50%)	8904 (62%)	10401 (57%)	<0.0001
Acetylsalicylic acid	7198 (40%)	5205 (37%)	5632 (31%)	<0.0001
Event rate in the control group	*N* = 6022	*N* = 7133	*N* = 9081	
Total stroke or SEE	202 (3.35%)	306 (4.29%)	265 (2.92%)	0.0001
Ischemic stroke	143 (2.37%)	226 (3.17%)	175 (1.93%)	<0.0001
Hemorrhagic stroke	45 (0.75%)	57 (0.80%)	78 (0.75%)	0.9968
SEE	16 (0.27%)	25 (0.35%)	15 (0.17%)	0.2367
Intracranial bleeding	90 (1.49%)	84 (1.18%)	122 (1.34%)	0.6421
Major bleeding	421 (6.99%)	386 (5.41%)	462 (5.09%)	<0.0001
Death	487 (8.09%)	632 (8.86%)	669 (7.37%)	0.0168
Treatment discontinuation^†^	1150 (19%)	2468 (35%)	2732 (30%)	<0.0001

*Chi-square test for categorical variables and one-way analysis of variance (ANOVA) for continuous variables.

^†^Treated patients that received assigned study drug but did not complete study.

CrCl: creatinine clearance; SEE: systemic embolic event; TTR: time in therapeutic range; VKA: vitamin K antagonist.

**Table 3:** Direct comparisons for main outcomes: absolute difference in events per 1000 patients treated per year and NNTB per year*.

*Population *	Non-hemorrhagic stroke and SEE		Intracranial bleeding	
Comparison	Risk difference (95% CI)	NNTB (95% CI)	Risk difference (95% CI)	NNTB (95% CI)
*All patients *				
All NOAC versus warfarin	−1 (−2.4 to 0.5)	1012 (NNTB 418 to ∞ to NNTH 2137)	−3.7 (−5.3 to −3.1)	271 (190 to 469)
Dabigatran versus warfarin	−1.1 (−3.6 to 1.4)	934 (NNTB 280 to ∞ to NNTH 700)	−4.9 (−6.5 to −3.2)	206 (153 to 316)
Rivaroxaban versus warfarin	−0.9 (−4.1 to 2.2)	1068 (NNTB 247 to ∞ to NNTH 458)	−2.1 (−3.8 to −0.4)	469 (263 to 2404)
Apixaban versus warfarin	−1.1 (−2.6 to 0.5)	940 (NNTB 388 to ∞ to NNTH 1984)	−4.3 (−5.9 to −2.7)	232 (168 to 364)

*Prior stroke or TIA *				
All NOAC versus warfarin	−0.4 (−3.8 to 3)	2404 (NNTB 263 to ∞ to NNTH 332)	−5.8 (−10.9 to −2.3)	173 (92 to 437)
Dabigatran versus warfarin	1.2 (−5.8 to 8.1)	853 (NNTB 174 to ∞ to NNTH 123)	−8.8 (−13.7 to −3.9)	113 (73 to 255)
Rivaroxaban versus warfarin	−0.5 (−5.3 to 4.3)	1923 (NNTB 189 to ∞ to NNTH 235)	−1.7 (−4.2 to 0.7)	583 (NNTB 240 to ∞ to NNTH 1479)
Apixaban versus warfarin	−1.7 (−8.9 to 5.5)	595 (NNTB 112 to ∞ to NNTH 180)	−8.2 (−12.9 to −3.5)	121 (77 to 283)

*No prior stroke or TIA *				
All NOAC versus warfarin	−1.1 (−2.7 to 0.4)	874 (NNTB 377 to ∞ to NNTH 2747)	−3.3 (−4.3 to −2.3)	305 (232 to 437)
Dabigatran versus warfarin	−1.6 (−4.2 to 1)	613 (NNTB 236 to ∞ to NNTH 1032)	−3.9 (−5.6 to −2.1)	258 (178 to 478)
Rivaroxaban versus warfarin	−1.8 (−5.7 to 2)	940 (NNTB 388 to ∞ to NNTH 1984)	−2.6 (−4.8 to −0.3)	392 (207 to 3846)
Apixaban versus warfarin	−0.7 (−3 to 1.5)	1374 (NNTB 337 to ∞ to NNTH 661)	−3.4 (−5 to −1.8)	293 (201 to 558)

*TTR ≥ 65% *				
All NOAC versus warfarin	−0.3 (−3.0 to 2.3)	3086 (NNTB 337 to ∞ to NNTH 441)	−4.5 (−6.4 to −2.6)	223 (156 to 384)
Dabigatran versus warfarin	1.1 (−2.1 to 4.3)	NNTH 934 (NNTB 467 to ∞ to NNTH 231)	−5.1 (−7.5 to −2.8)	196 (134 to 363)
Rivaroxaban versus warfarin	NA	NA	−3.4 (−6.8 to −0.1)	296 (148 to 19231)
Apixaban versus warfarin	−1.6 (−4.5 to 1.4)	638 (NNTB 220 to ∞ to NNTH 714)	NA	NA

*TTR < 65% *				
All NOAC versus warfarin	−3.5 (−6.3 to −0.8)	283 (159 to 1190)	−2.9 (−5.7 to −0.2)	344 (177 to 6536)
Dabigatran versus warfarin	−3.2 (−6.9 to 0.6)	316 (NNTB 144 to ∞ to NNTH 1751)	−4.4 (−6.9 to −1.9)	228 (145 to 516)
Rivaroxaban versus warfarin	NA	NA	−1.7 (−3.6 to 0.3)	601 (NNTB 279 to ∞ to NNTH 3205)
Apixaban versus warfarin	−3.6 (−7.2 to 0.1)	279 (NNTB 138 to ∞ to NNTH 17857)	NA	NA

*Random effects model, intention-to-treat.

NA: data not available; NNTB: number of patients needed to be treated for one additional patient to benefit; NNTH: number of patients needed to be treated for one additional patient to be harmed; NOAC: new oral anticoagulants; SEE: systemic embolic event; TIA: transient ischemic attack; TTR: time in therapeutic range.

**Table 4:** Direct comparisons for secondary outcomes: absolute difference in events 1000 patients treated per year and NNTB per year*.

*Population *	All strokes and SEE		Major bleeding	
Comparison	Risk difference (95% CI)	NNTB (95% CI)	Risk difference (95% CI)	NNTB (95% CI)
*All patients *				
All NOAC versus warfarin	−3.2 (−4.8 to −1.6)	310 (207 to 620)	−4 (−9 to 1)	253 (NNTB 111 to ∞ to NNTH 962)
Dabigatran versus warfarin	−3.7 (−6.5 to −1)	269 (154 to 980)	−4.4 (−8.4 to −0.5)	228 (120 to 2179)
Rivaroxaban versus warfarin	−2.7 (−6 to 0.7)	370 (NNTB 166 to ∞ to NNTH 1479)	0.7 (−3.2 to 4.5)	NNTH 1479 (NNTB 310 to ∞ to NNTH 221)
Apixaban versus warfarin	−3.3 (−5.9 to −0.7)	303 (168 to 1374)	−8.4 (−11.7 to −5.1)	119 (85 to 196)

*European population *				
All NOAC versus warfarin	−0.9 (−3.1 to 1.4)	1131 (NNTB 321 to ∞ to NNTH 712)	−3.0 (−5.7 to −0.3)	337 (176 to 3846)
Dabigatran versus warfarin	−0.6 (−4.6 to 3.5)	1783 (NNTB 218 to ∞ to NNTH 288)	−3.2 (−8.4 to 2.1)	316 (NNTB 119 to ∞ to NNTH 467)
Rivaroxaban versus warfarin	−2.3 (−7 to 2.3)	437 (NNTB 145 to ∞ to NNTH 437)	−2.1 (−6.7 to 2.3)	469 (NNTB 150 to ∞ to NNTH 427)
Apixaban versus warfarin	−0.3 (−4 to 3.4)	2976 (NNTB 252 to ∞ to NNTH 298)	−3.8 (−8.4 to 0.9)	263 (NNTB 119 to ∞ to NNTH 1276)

*Non-European population *				
All NOAC versus warfarin	−4.9 (−7.1 to −2.7)	205 (140 to 377)	−4.3 (−12.5 to 3.9)	232 (NNTB 80 to ∞ to NNTH 256)
Dabigatran versus warfarin	−5.7 (−9.3 to −2)	177 (108 to 503)	−5.2 (−10.7 to 0.3)	192 (NNTB 94 to ∞ to NNTH 3922)
Rivaroxaban versus warfarin	−3.1 (−8.1 to 1.9)	321 (NNTB 123 to ∞ to NNTH 534)	4 (−2.6 to 10.5)	NNTH 253 (NNTB 385 to ∞ to NNTH 95)
Apixaban versus warfarin	−5.4 (−9 to −1.8)	186 (112 to 558)	−11.5 (−16.1 to −6.9)	87 (62 to 144)

*Prior stroke or TIA *				
All NOAC versus warfarin	−3.6 (−7.4 to 0.1)	275 (NNTB 135 to ∞ to NNTH 19231)	−4.1 (−8.7 to 0.6)	247 (NNTB 114 to ∞ to NNTH 1603)
Dabigatran versus warfarin	−5.5 (−13.2 to 2.3)	183 (NNTB 76 to ∞ to NNTH 436)	−6.3 (−15.8 to 3.1)	158 (NNTB 63 to ∞ to NNTH 321)
Rivaroxaban versus warfarin	−1.4 (−6.5 to 3.7)	712 (NNTB 154 to ∞ to NNTH 271)	−1 (−6 to 4.1)	1012 (NNTB 166 to ∞ to NNTH 243)
Apixaban versus warfarin	−7.4 (−15.5 to 0.7)	135 (NNTB 64 to ∞ to NNTH 1374)	−8.6 (−17 to −0.2)	116 (59 to 4464)

*No prior stroke or TIA *				
All NOAC versus warfarin	−2.9 (−4.6 to −1.2)	350 (219 to 836)	−3.5 (−9.1 to 2)	283 (NNTB 111 to ∞ to NNTH 506)
Dabigatran versus warfarin	−3.3 (−6.2 to −0.5)	302 (162 to 1961)	−3.9 (−3.2 to 0.4)	255 (NNTB 316 to ∞ to NNTH 2451)
Rivaroxaban versus warfarin	−4.3 (−8.5 to 0)	235 (117 to ∞)	2.6 (−3.4 to 8.5)	NNTH 392 (NNTB 291 to ∞ to NNTH 118)
Apixaban versus warfarin	−2.2 (−4.8 to 3.0)	446 (NNTB 208 to ∞ to NNTH 2976)	−8.3 (−11.9 to −0.5)	121 (84 to 213)

*TTR ≥ 65% *				
All NOAC versus warfarin	−2.6 (−4.8 to −0.4)	385 (207 to 2404)	−0.6 (−6.6 to 7.7)	NNTH 1748 (NNTB 153 to ∞ to NNTH 130)
Dabigatran versus warfarin	−2 (−5.7 to 1.6)	490 (NNTB 177 to ∞ to NNTH 633)	0.1 (−5.4 to 5.7)	NNTH 9804 (NNTB 185 to ∞ to NNTH 177)
Rivaroxaban versus warfarin	−4.2 (−9.7 to 1.3)	240 (NNTB 103 to ∞ to NNTH 769)	9.1 (0.2 to 17.9)	NNTH 111 (56 to 4808)
Apixaban versus warfarin	−2.7 (−6.1 to 0.7)	372 (NNTB 165 to ∞ to NNTH 1488)	−5.4 (−10.4 to −0.4)	186 (97 to 2551)

*TTR < 65% *				
All NOAC versus warfarin	−4 (−6.2 to −1.9)	250 (162 to 534)	−7.1 (−12.7 to −1.6)	140 (78 to 641)
Dabigatran versus warfarin	−5.3 (−9.4 to −1.2)	189 (107 to 853)	−8.8 (−14.4 to −3.2)	113 (69 to 316)
Rivaroxaban versus warfarin	−3.3 (−6.9 to 0.2)	300 (NNTB 146 to ∞ to NNTH 6410)	−2 (−6.3 to 2.3)	493 (NNTB 159 to ∞ to NNTH 437)
Apixaban versus warfarin	−4 (−8 to 0)	252 (126 to ∞)	−11.4 (−15.8 to −7)	88 (63 to 143)

*Random effects model, intention-to-treat.

NA: data not available; NNTB: number of patients needed to be treated for one additional patient to benefit; NNTH: number of patients needed to be treated for one additional patient to be harmed; NOAC: new oral anticoagulants; SEE: systemic embolic event; TIA: transient ischemic attack; TTR: time in therapeutic range.
