# Supplementary material for: Dabigatran, Rivaroxaban, or Apixaban versus Warfarin in Patients with Nonvalvular Atrial Fibrillation: A Systematic Review and Meta-Analysis of Subgroups
Source: Thrombosis. 2013 Dec 22;2013:640723. doi: 10.1155/2013/640723 (PMC3885278; doi:10.1155/2013/640723)
Supplement: Supplementary file 1 — Supplementary Material includes details of the bibliographic search, as well as risk of bias assessment for the included studies, characteristics of the patients and events in patients with prior stroke or transient ischemic attack, sensitivity analyses depending on statistical model used and risk of bias, and complete subgroup analyses for main and key secondary outcomes. [file 640723.f1.doc]

**SUPPLEMENTARY APPENDIX:**

**DETAILS OF THE BIBLIOGRAPHIC SEARCH:**

**1) Main bibliographic search:**

**1a. MEDLINE:**

http://www.ncbi.nlm.nih.gov/pubmed

**Search:** “Terms: rivaroxaban and/or dabigatran and/or apixaban; “Limits Activated: Publication Date: from 2000/01/01 to 2012/12/31.”

**Results: 1561** articles.

**1b. CENTRAL:**

http://onlinelibrary.wiley.com/o/cochrane/cochrane_clcentral_articles_fs.html

**Search:** “Terms: rivaroxaban or dabigatran or apixaban (Search Text Fields).”; “Limits Activated: Data range from 2000 to 2012.

**Results:** **138** articles (no additional clinical trials to those identified through MEDLINE were found).

**2) Additional bibliographic search:**

**2a. Regulatory Authorities:**

**Food and Drug Administration (FDA):**

www.accessdata.fda.gov/scripts/cder/drugsatfda

**European Medicines Agency (EMA):**

www.ema.europa.eu

**2b. Clinical trials websites:**

**Clinicaltrials.gov Registry (“U.S. National Institute of Health”):**

http://www.clinicaltrials.gov/

**EU Clinical Trials Register**

https://www.clinicaltrialsregister.eu/ctr-search/search

**Boehringer-Ingelheim clinical trial register website (dabigatran studies):**

http://trials.boehringer-ingelheim.com/trial_results.html

**Bayer Healthcare clinical trial register website (rivaroxaban studies):**

http://healthcare.bayer.com/scripts/pages/en/research_development/clinical_trials/trial_finder/index.php

**2c. Abstracts from international congresses:**

**American Society of Hematology (ASH):**

**2007:** http://abstracts.hematologylibrary.org/content/vol110/issue11/

**2008:** http://abstracts.hematologylibrary.org/content/vol112/issue11/

**2009:** http://abstracts.hematologylibrary.org/content/vol114/issue22/

**2010:** http://abstracts.hematologylibrary.org/content/vol116/issue21/

**2011:** http://abstracts.hematologylibrary.org/content/vol118/issue21/

**2012:** http://abstracts.hematologylibrary.org/content/vol120/issue21/

**American College of Cardiology (ACC) Annual Scientific Sessions:**

**2007-2010:** http://content.onlinejacc.org/

**2011-2012:** http://www.cardiosource.org/Meetings/Previous-Meetings-OLD.aspx

**American Heart Association (AHA) Scientific Sessions:**

**2007:** http://circ.ahajournals.org/content/vol116/16_MeetingAbstracts/

**2008:** http://circ.ahajournals.org/content/vol118/18_MeetingAbstracts/

**2009:** http://circ.ahajournals.org/content/vol120/18_MeetingAbstracts/

**2010:** http://circ.ahajournals.org/content/vol122/21_MeetingAbstracts/

**2011:** http://circ.ahajournals.org/content/vol124/21_MeetingAbstracts/

**2012:** http://www.abstractsonline.com/Plan/start.aspx

**European Society of Cardiology (ESC)**

**2007:** http://www.escardio.org/congresses/esc2007/Pages/resources.aspx

**2008:** http://www.escardio.org/congresses/esc2008/Pages/welcome.aspx

**2009:** http://www.escardio.org/congresses/esc-2009/Pages/welcome.aspx

**2010:** http://www.escardio.org/congresses/esc-2010/Pages/welcome.aspx

**2011:** http://www.escardio.org/congresses/esc-2011/Pages/welcome.aspx

**2012:** http://www.escardio.org/congresses/esc-2012/Pages/welcome.aspx

**Table A1 Risk of bias for the 3 included studies**

| **STUDY/Characteristic** | **Judgement** | **Support for judgement** |
| --- | --- | --- |
| **RE-LY** |  |  |
| Random sequence generation (selection bias) | Low risk. | Quote: “all trial participants were randomly assigned”. |
| Allocation concealment (selection bias) | Low risk. | Quote: “central, interactive, automated telephone system”. |
| Blinding of participants and personnel (performance bias) | Unclear risk. | Quote: “multicenter, prospective, open-label, randomized trial, with blinded adjudication of all outcomes (PROBE design)”.  Comment: There were multiple measures put in place to ensure the robustness and reliability of the results: a) data management was external to the sponsor and was managed by an independent academic group with firewalls to protect the integrity of the study; b) adjudication was performed with procedures to ensure adjudicators were unaware of treatment group assignments and the selected outcomes were of clinical importance; c) stroke and bleeding questionnaires were used at every visit to decrease ascertainment bias and subjects in all treatment groups had a similar number of visits to the study centers; d) screening of free text fields on case report form (CRF) were done to identify subjects with findings that could be indicative of an outcome event.; e) the study was blinded for dabigatran doses. However, these measures cannot remove all the bias of an open-label design [FDA briefing]. |
| Blinding of outcome assessment (detection bias) | Unclear risk. | Quote: “The outcome events including strokes, non-central nervous system systemic emboli, deaths, myocardial infarctions, pulmonary embolism, major bleeds, and some minor bleeds are adjudicated by a blinded adjudication committee”. “An independent data safety monitoring board reviewed the unblinded study data and performed two prespecified interim analyses of efficacy.”  Comment: The potential influence of the 2 unblinded interim analyses on study conduct is unknown. |
| Incomplete outcome data addressed (attrition bias) | Unclear risk. | Insufficient reporting of attrition/exclusions to permit judgement of ‘Low risk’ or ‘High risk’. There were higher withdrawal rates in patients on dabigatran and “as-treated” analysis is not publicly available. |
| Selective reporting (reporting bias) | Unclear risk. | The study protocol is available and all of the study’s pre-specified (primary and secondary) outcomes that are of interest in the review have been reported in the pre-specified way. However, in an open-label study there may have been biases when it came to investigator reporting of adverse event as well as when investigators chose to discontinue study medication [FDA briefing]. |
| **Summary** | **Unclear risk** | **Unclear risk of bias for one or more key domains.** |
| **ROCKET-AF** |  |  |
| Random sequence generation (selection bias) | Low risk. | Quote: “patients were randomly allocated.” |
| Allocation concealment (selection bias) | Low risk. | Quote: “To effect concealment of randomization, treatment allocation is randomized using a blinded, central telephonic Interactive Voice Response System” |
| Blinding of participants and personnel (performance bias) | Low risk. | Quote: “A double-blind design was chosen to minimize bias in cointerventions and interpretation of clinical events. To maintain blinding in ROCKET AF, sham INR results were provided. Patients in each group also received a placebo tablet in order to maintain blinding”. |
| Blinding of outcome assessment (detection bias) | Low risk. | Quote: “The independent blinded clinical end-point committee…applied protocol definitions to adjudicate all suspected cases of stroke, systemic embolism, myocardial infarction, death, and bleeding events that contributed to the prespecified end points” |
| Incomplete outcome data addressed (attrition bias) | Low risk. | Reasons for missing outcome data unlikely to be related to true outcome; Missing outcome data balanced in numbers across intervention groups, with similar reasons for missing data across groups; The proportion of missing outcomes compared with observed event risk not enough to have a clinically relevant impact on the intervention effect estimate. |
| Selective reporting (reporting bias) | Low risk. | The study protocol is available and all of the study’s pre-specified (primary and secondary) outcomes that are of interest in the review have been reported in the pre-specified way; |
| **Summary** | **Low risk** | **Low risk of bias for all key domains.** |
| **ARISTOTLE** |  |  |
| Random sequence generation (selection bias) | Low risk. | Quote: “patients were randomly assigned.” |
| Allocation concealment (selection bias) | Low risk. | Quote: “central response system.” |
| Blinding of participants and personnel (performance bias) | Low risk. | Quote: “double-blind”. To maintain blinding, study medications are packaged using a double-dummy design. INRs were monitored with the use of a blinded, encrypted, point-of-care INR device”. |
| Blinding of outcome assessment (detection bias) | Low risk. | Quote: “The primary and secondary efficacy and safety outcomes were adjudicated on the basis of prespecified criteria by a clinical-events committee whose members were not aware of study-group assignments”. |
| Incomplete outcome data addressed (attrition bias) | Low risk. | Reasons for missing outcome data unlikely to be related to true outcome; Missing outcome data balanced in numbers across intervention groups, with similar reasons for missing data across groups; The proportion of missing outcomes compared with observed event risk not enough to have a clinically relevant impact on the intervention effect estimate. |
| Selective reporting (reporting bias) | Low risk. | The study protocol is available and all of the study’s pre-specified (primary and secondary) outcomes that are of interest in the review have been reported in the pre-specified way. |
| **Summary** | **Low risk** | **Low risk of bias for all key domains.** |

**Table A2** Characteristics of the patients and events in patients with prior stroke or transient ischemic attack

| **Drug, trial** | **Dabigatran**  **RE-LY^22,29^** | **Rivaroxaban**  **ROCKET^23,36^** | **Apixaban**  **ARISTOTLE^24,37^** | **p-value*** |
| --- | --- | --- | --- | --- |
|  | N=3623 | N=7468 | N=3436 |  |
| **Patients characteristics** |  |  |  |  |
| Age (years) | 70.4 (mean) | 71 (median) | 70.1 (mean) | - |
| Male gender | 2279 (63%) | 4538 (61%) | 2152 (63%) | 0.1825 |
| CHADS_2_  (mean or median) | - | 3 (median) | 3.7 (mean) | - |
| CHADS_2_ ≥3 | 3246 (90%) | 7044 (94%) | 3168 (92%) | **<0.0001** |
| CHADS_2_ ≥2 | 3623 (100%) | 7468 (100%) | 3436 (100%) | - |
| CHADS_2_ =1 | 0 | 0 | 0 | - |
| Congestive heart failure | - | 3785 (51%) | 939 (27%) | - |
| Hypertension | 2783 (77%) | 6343 (85%) | 2858 (83%) | **<0.0001** |
| Age ≥75 years | - | - | 1205 (35%) | - |
| Diabetes | 816 (23%) | 1806 (24%) | 902 (26%) | **0.0095** |
| Prior myocardial infarction | - | 1131 (15%) | 587 (17%) |  |
| TTR prior stroke, median % (range) | 63 (mean) | 57 (43-70) | 65 (51-76) |  |
| TTR no prior stroke, median % (range) | 65 (mean) | 59 (44-71) | 66 (53-77) |  |
| p-value (TTR prior stroke versus no prior stroke) | Not reported | **0.041** | **0.022** |  |
| **Type of atrial fibrillation** |  |  |  |  |
| Permanent-persistent | - | 5924 (79%) | - | - |
| Paroxysmal | - | 1437 (19%) | - | - |
| **Antithrombotic treatment at baseline** |  |  |  |  |
| VKA | 2009 (55%) | 4429 (59%) | 2082 (61%) | **0.0002** |
| Acetylsalicylic acid | 1444 (40%) | 2808 (38%) | 1067 (31%) | **<0.0001** |
| **Event rate in the control group** | N=1195 | N=3714 | N=1742 |  |
| Total stroke or SEE | 65 (5.44%) | 187 (5.04%) | 98 (5.63%) | 0.9215 |
| Ischemic stroke | 41 (3.43%) | 144 (3.88%) | 68 (3.90%) | 0.9678 |
| Hemorrhagic stroke | 18 (1.51%) | 30 (0.81%) | 31 (1.78%) | **0.0289** |
| Systemic embolism | 6 (0.50%) | 17 (0.46%) | 2 (0.11%) | 0.2615 |
| Intracranial bleeding | 30 (2.51%) | 46 (1.24%) | 41 (2.35%) | **0.0099** |
| Major bleeding | 97 (8.12%) | 183 (4.93%) | 106 (6.08%) | **0.0018** |
| Death from any cause | 107 (8.95%) | 294 (7.92%) | 150 (8.61%) | 0.8065 |
| Treatment discontinuation | - | - | - | - |

SEE = systemic embolic event; TTR = time in therapeutic range; VKA = vitamin K antagonist.

*Chi-square test for categorical variables and one-way analysis of variance (ANOVA) for continuous variables

**Table A3** Sensitivity analyses depending on statistical model used and risk of bias

| **Outcome** | **Base case:**  **random effects model, all studies** | **Sensitivity analysis 1:**  **fixed effects model, all studies** | **Sensitivity analysis 2:**  **random effects, studies at low risk of bias*** |
| --- | --- | --- | --- |
|  | **Relative risk (95%CI)** | **Relative risk (95%CI)** | **Relative risk (95%CI)** |
| **Non-hemorrhagic stroke and systemic embolic event** | 0.93 (0.83 to 1.04) | 0.93 (0.83 to 1.04) | 0.94 (0.82 to 1.07) |
| **Intracranial bleeding** | 0.46 (0.33 to 0.65) | 0.46 (0.38 to 0.56) | 0.53 (0.34 to 0.80) |
| **All strokes and systemic embolic events** | 0.82 (0.74 to 0.91) | 0.82 (0.74 to 0.91) | 0.84 (0.75 to 0.95) |
| **Major bleeding** | 0.86 (0.70 to 1.05) | 0.86 (0.80 to 0.93) | 0.85 (0.59 to 1.22) |
| **Deaths** | 0.91 (0.85 to 0.97) | 0.91 (0.85 to 0.96) | 0.91 (0.84 to 0.98) |

*ROCKET-AF and ARISTOTLE

**Figure A1** Subgroup analyses for non-hemorrhagic stroke and systemic embolic events

**Figure A2** Subgroup analyses for intracranial bleeding

**Figure A3** Subgroup analyses for all strokes and systemic embolic events

**Figure A3** Subgroup analyses for all strokes and systemic embolic events (continued)

**Figure A3** Subgroup analyses for all strokes and systemic embolic events (continued)

**Figure A3** Subgroup analyses for all strokes and systemic embolic events (continued)

**Figure A3** Subgroup analyses for all strokes and systemic embolic events (continued)

**Figure A4** Subgroup analyses for major bleeding

**Figure A4** Subgroup analyses for major bleeding (continued)

**Figure A4** Subgroup analyses for major bleeding (continued)

**Figure A4** Subgroup analyses for major bleeding (continued)

**Figure A4** Subgroup analyses for major bleeding (continued)

**Figure A5** Subgroup analyses for deaths
